# Strongly absorbing molecules make tissue transparent: a new insight for understanding tissue optical clearing

**DOI:** 10.1038/s41377-024-01675-z

**Published:** 2025-01-01

**Authors:** Tingting Yu, Dan Zhu

**Affiliations:** Britton Chance Center for Biomedical Photonics-MoE Key Laboratory for Biomedical Photonics, Advanced Biomedical Imaging Facility-Wuhan National Laboratory for Optoelectronics, Huazhong University of Science and Technology, Wuhan, 430074 Hubei China

**Keywords:** Optical techniques, Other photonics

## Abstract

Optical imaging plays a central role in the field of biomedicine, but it suffers from the light scattering of tissues. The research group from Stanford University has reported a counterintuitive observation that strongly absorbing molecules could achieve optical transparency in live animals, providing a new insight for understanding tissue optical clearing. It empowers scientists to leverage optical imaging techniques for in vivo observation of a wide range of deep-seated structures and activities.

The latest breakthrough in the field of live tissue optical clearing reported in *Science* is attracting more and more attentions^1^. The new insight promises to enhance our comprehension of the underlying principles and to provide new perspective for developing innovative methods in tissue optical clearing.

Optical imaging provides a powerful tool for exploring life science. However, the inherent scattering and absorption properties of biological tissues significantly impede the penetration of light, leading to a decline in imaging resolution and contrast as it propagates deeper. Scientists have been making efforts on solving this problem by developing innovative contrast agents and employing cutting-edge imaging techniques^2^. Typically, researchers have relied on mechanical sectioning to reconstruct 3D structures of large tissues or organs, or established window models for in vivo imaging to peer deeper into biological systems. If the body were transparent, these tasks would be a breeze. It may sound like science fiction, but some have already succeeded^3^. For instance, Richard White used careful breeding techniques to create a transparent adult zebrafish^4^, which has been applied to study cancer development and neuroscience. Unfortunately, to date, no other transparent animal species have been artificially created. Tissue optical clearing emerged as a more promising avenue for deep tissue imaging. Over the past few years, our lab has been at the forefront of the in vivo clearing field, inventing various in vivo skin optical clearing methods and pioneering the development of skull optical clearing windows^5–13^. These advancements have facilitated the observation and manipulation of cutaneous or cortical neuro-vascular structure and function in deep^14–17^. We have also made some efforts in ex vivo clearing techniques for whole-organ profiling^18–21^, a field that has been flourishing over the last two decades^22–24^.

Light scattering in tissue occurs due to the difference between low refractive index (RI) aqueous-based components (such as interstitial fluid and cytosol) and high RI lipid-and protein-based components (such as the plasma membrane, myelin, and myofibrils)^25^. Existing tissue clearing methods typically enhance RI matching by substituting water with chemicals of a higher RI, generally accompanied by procedures like dehydration, decalcification, delipidation, and collagen dissociation to yield a homogeneous environment^18–20^. Alternatively, Yasuhiko et al. have introduced an inspiring in-silico clearing to realize deep RI tomography and improved deep imaging capability by partial reconstruction and wave-backpropagation^26^. For pigment-rich tissues, such as the liver, spleen, eyes, and others, a decolorization procedure is necessary to reduce the tissue absorption and thereby improve imaging performance^21^.

Recently, Ou et al. reported on a counterintuitive study that strongly absorbing molecules could achieve optical transparency in live biological tissues^1^, providing a new insight for understanding tissue optical clearing (Fig. 1). They found that tartrazine, an aqueous solution of a common food color approved by the US FDA, can reversibly make the skin, muscle, and connective tissues transparent in live rodents. This allows for the visualization of a wide range of deep-seated structures and activities. Concurrently, they made a new breakthrough of tissue clearing from fundamental optical concepts in textbook of optics and revealed the physical mechanism behind this phenomenon. As known, the complex RI is composed of the real and imaginary components, with the former relating to dispersion or scattering and the latter associated with absorption, and their spectral dependences can be connected through causality as per the Kramers-Kronig relations. The authors used the Lorentz oscillator model, in conjunction with the Kramers-Kronig relations, to predict the tissue transparency that the molecules can achieve. They revealed that Lorentzian oscillators with sharp absorption resonances in the visible spectrum (400–750 nm) are very effective in raising the real part of the RI in the red and infrared parts of the spectrum when dissolved in water, indicating that water-soluble dyes in the visible spectrum hold the potential to effectively reduce the RI mismatching of tissue components and achieve optical transparency.

**Fig. 1:**
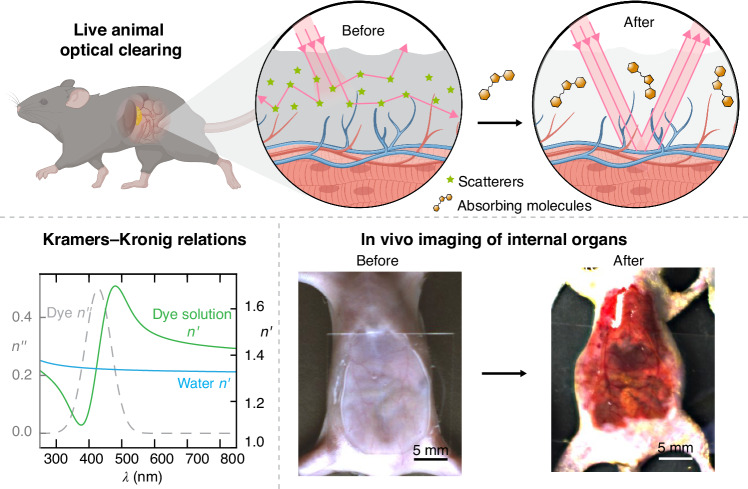
Live animal optical clearing with absorbing dye molecules^1^

The remarkable transparency achieved, attainable through a simple topical application of dye solution, empowers scientists to leverage a spectrum of optical techniques for in vivo observation of internal or deep tissues through the scalp, abdomen, and leg skin, indicating broad potential for applications. As illustrated in the original paper, this has facilitated high-resolution imaging of abdominal organs, such as the small intestine and liver, without the need for abdominal surgery, which is rather impressive. This approach minimizes the reliance on invasive surgical window creation for deep tissue imaging, and is anticipated to unlock new perspectives across a multitude of research disciplines. People can also directly observe what’s going on inside the organism with the naked eye, which has the potential to revolutionize existing optical research paradigm in life science. In the future, the development of this technology holds promise for significantly enhancing the diagnostic potential of certain imaging techniques. For instance, speckle imaging technology, currently used to monitor superficial skin vascular abnormalities, could be augmented to detect and monitor vascular complications in the lower limbs associated with diabetes, thanks to the advancements in tissue transparency and imaging clarity.

It is noted that the current study was conducted on young mice, not humans. Human skin is almost ten times thicker than that of mice, and there is uncertainty regarding the amount of dye required to effectively penetrate such thickness. Therefore, the validity of tartrazine in clearing tissue will be a challenge in larger organisms and humans. The exploration of dyes that have a stronger clearing effect than tartrazine remains to be done. In any case, it is very impressive that people with an optical background know the formula of complex RI, but most of them only consider the scattering in terms of the real component. Fortunately, Ou et al. paid attention to the imaginary component of the RI, using strongly absorbing dyes to enhance the RI of aqueous solution to clear live tissues. This theoretical framework may open a new perspective on developing optical clearing methods, encompassing both in vivo and ex vivo clearing methodologies. This is a vivid exercise in using basic optical knowledge to create new technologies, which could bring us another significant enlightenment beyond the research itself.
